# Synergy between pluripotent stem cell‐derived macrophages and self‐renewing macrophages: Envisioning a promising avenue for the modelling and cell therapy of infectious diseases

**DOI:** 10.1111/cpr.13770

**Published:** 2024-11-13

**Authors:** Dingkun Peng, Meilin Li, Zhuoran Yu, Tingsheng Yan, Meng Yao, Su Li, Zhonghua Liu, Lian‐Feng Li, Hua‐Ji Qiu

**Affiliations:** ^1^ State Key Laboratory for Animal Disease Control and Prevention, Harbin Veterinary Research Institute Chinese Academy of Agricultural Sciences Harbin China; ^2^ Key Laboratory of Animal Cellular and Genetic Engineering of Heilongjiang Province, College of Life Science Northeast Agricultural University Harbin China

**Keywords:** differentiation, infectious diseases, macrophages, pluripotent stem cells, self‐renewal

## Abstract

As crucial phagocytes of the innate immune system, macrophages (M*ϕ*s) protect mammalian hosts, maintain tissue homeostasis and influence disease pathogenesis. Nonetheless, M*ϕ*s are susceptible to various pathogens, including bacteria, viruses and parasites, which cause various infectious diseases, necessitating a deeper understanding of pathogen–M*ϕ* interactions and therapeutic insights. Pluripotent stem cells (PSCs) have been efficiently differentiated into PSC‐derived M*ϕ*s (PSCdM*ϕ*s) resembling primary M*ϕ*s, advancing the modelling and cell therapy of infectious diseases. However, the mass production of PSCdM*ϕ*s, which lack proliferative capacity, relies on large‐scale expansions of PSCs, thereby increasing both costs and culture cycles. Notably, M*ϕ*s deficient in the *MafB/c‐Maf* genes have been reported to re‐enter the cell cycle with the stimulation of specific growth factor cocktails, turning into self‐renewing M*ϕ*s (SRM*ϕ*s). This review summarizes the applications of PSCdM*ϕ*s in the modelling and cell therapy of infectious diseases and strategies for establishing SRM*ϕ*s. Most importantly, we innovatively propose that PSCs can serve as a gene editing platform to creating PSC‐derived SRM*ϕ*s (termed PSRM*ϕ*s), addressing the resistance of M*ϕ*s against genetic manipulation. We discuss the challenges and possible solutions in creating PSRM*ϕ*s. In conclusion, this review provides novel insights into the development of physiologically relevant and expandable M*ϕ* models, highlighting the enormous potential of PSRM*ϕ*s as a promising avenue for the modelling and cell therapy of infectious diseases.

## Graphical abstract

This review highlights the use of pluripotent stem cell‐derived macrophages (PSCdM*ϕ*s) in modelling and treating infectious diseases and explores methods to create self‐renewing macrophages (SRM*ϕ*s). We further propose that pluripotent stem cells can be utilized as a gene editing platform to develop self‐renewing macrophages (PSRM*ϕ*s).

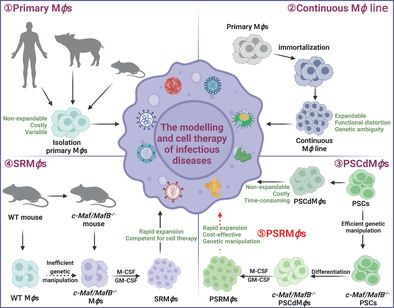

## 1 INTRODUCTION

Pathogens such as bacteria, viruses and parasites can infect macrophages (M*ϕ*s) to cause various infectious diseases in humans and animals by evading the immune response and using M*ϕ*s as ‘Trojan horses’ for transmission.^1^, ^2^ The pathogen–M*ϕ* interactions are intricately complex, in which M*ϕ*s exert varied roles in different scenarios. For instance, African swine fever virus (ASFV) primarily replicates in M*ϕ*s,^3^ *Chlamydia trachomatis* hijacks M*ϕ*s as reservoirs^4^ and influenza A virus (IAV) is contained by alveolar M*ϕ*s (AM*ϕ*s).^5^ Furthermore, various pathogens have developed several sophisticated strategies to evade the immune responses of M*ϕ*s. For example, *Mycobacterium tuberculosis* avoids destruction by preventing the fusion of phagosomes with lysosomes,^6^ while human immunodeficiency virus (HIV) conceals its replication processes through specific cofactor recruitment, thereby evading innate immune recognition.^7^ Therefore, understanding pathogen–M*ϕ* interactions is crucial for preventing and controlling infectious diseases. However, the progress has been hampered by the unavailability of suitable M*ϕ* models. Primary M*ϕ*s, such as peripheral blood monocyte‐derived M*ϕ*s (MDMs) and bone marrow‐derived M*ϕ*s (BMDMs), exhibit authentic phenotypes with their in vivo counterparts but display various limitations, such as challenge in proliferation, high cost‐consumption and batch‐to‐batch variation.^8^, ^9^ Continuous cell lines such as THP‐1, RAW264 and J774.A.1 are widely used but suffer from functional distortion and genetic ambiguity.^10^, ^11^ Notably, both primary and continuous M*ϕ*s are resistant to genetic modification due to the presence of host restriction factors,^12^, ^13^ highlighting the need for a suitable M*ϕ* models for investigating infectious diseases.

Pluripotent stem cells (PSCs), including embryonic stem cells (ESCs) and induced pluripotent stem cells (iPSCs), are valuable tools for developmental biology, disease modelling and organoid research.^14^, ^15^, ^16^, ^17^ PSCs can be differentiated into various cell types of interest due to their infinite self‐renewal capacity and pluripotency, thus providing diverse cell resources. To date, PSC‐derived M*ϕ*s (PSCdM*ϕ*s) with normal phenotypes and functions have been developed in vitro by multiple protocols.^18^, ^19^ Importantly, the compatibility of PSCs with genetic manipulation makes it feasible to obtain genetically modified PSCdM*ϕ*s, which is essential for studying pathogen–PSCdM*ϕ* interactions. However, similar to primary M*ϕ*s, PSCdM*ϕ*s lose the proliferation ability of PSCs. Fortunately, contrary to conventional wisdom, primary M*ϕ*s can re‐enter cell cycle without transformation under special circumstances, leading to the formation of self‐renewing M*ϕ*s (SRM*ϕ*s).^20^ Mechanistically, a stem cell‐like self‐renewal gene network in M*ϕ*s is controlled by M*ϕ*‐specific enhancer reservoir, and the genetic disruption of key genes activates the network and creates SRM*ϕ*s.^21^, ^22^ The SRM*ϕ*s are reported focus on mice, likely due to the resistance of M*ϕ*s to gene editing, which may account for the restricted application of SRM*ϕ*s. Interestingly, PSCs hold promise as a gene editing platform for M*ϕ*s, providing new insights into the SRM*ϕ* generation.

Here, we summarize the applications of PSCdM*ϕ*s in the modelling and cell therapy of infectious diseases, along with different strategies for creating SRM*ϕ*s. Importantly, we propose a promising avenue for the modelling and cell therapy of infectious diseases by using PSCs as a platform for genetic manipulation to generate PSC‐derived SRM*ϕ*s (PSRM*ϕ*s) with potential challenges and solutions discussed.

## 2 PSCdM*ϕ*s revolutionize the modelling and cell therapy of infectious diseases

To date, significant strides have been made in generating PSCdM*ϕ*s with similar phenotypes and functions to primary M*ϕ*s, including the expression of lineage markers, phagocytosis and the ability to release inflammatory factors.^23^ These PSCdM*ϕ*s also retain the polarization plasticity triggered by lipopolysaccharide (LPS) and interferon‐gamma (IFN‐*γ*) or IL‐4.^24^ These attributes underscore the utility of PSCdM*ϕ*s as models for exploring immune responses and M*ϕ* functions. Here, we summarize the protocols for the generation of PSCdM*ϕ*s and emphasize the applications of PSCdM*ϕ*s in the modelling and cell therapy of infectious diseases, which can be divided into three aspects: pathogen–PSCdM*ϕ* interactions, drug screening and testing and PSCdM*ϕ* based cell therapy (Table 1).

**TABLE 1:** PSCdM*ϕ*s for the modelling and cell therapy of infectious diseases.

Pathogens	Pluripotent stem cell lines	Differentiation protocols	Research findings	References
ZIKV (DENV)	hESCs hiPSCs	EB‐S	DENV infection in M*ϕ*s triggers MIF secretion and hampers migration, whereas ZIKV infection prolongs migration and suppresses proinflammatory responses.	25
	hiPSCs	EB‐F	PSCdMGs infected with ZIKV rather than DENV exhibits low cytotoxicity and penetrates neural organoids, spreading ZIKV to neural tissue.	26
	hiPSCs	EB‐F	IFNAR2 deficient in PSCdM*ϕ*s resulted in a notable increase in ZIKV replication and cell death.	27
HIV‐1	hiPSCs	EB‐F	*CCR5*‐mutated PSCdM*ϕ*s are resistant to HIV‐1 challenge.	28
	hiPSCs	OP9‐C	PSCdM*ϕ*s derived from *CCR5*‐modified iPSCs exhibit resistance to *CCR5*‐tropic virus infection.	29
	hiPSCs	2D‐F	PSCdM*ϕ*s derived from *CCR5*‐edited iPSCs are resistant to distinct HIV‐1 strains.	30
	NHP iPSCs	OP9‐C	PSCdM*ϕ*s derived from *CCR5*‐edited NHP iPSCs are resistant to CCR5‐tropic SIV strain.	31
	hiPSCs	2D‐F	PSCdM*ϕ*s derived from *CCR5Δ32* iPSCs show similar phenotypes and functions to wild‐type individuals but resistance to HIV infection.	32
	hiPSCs	OP9‐C	PSCdM*ϕ*s derived from iPSCs expressing anti‐HIV genes are resistant to HIV‐1 infection.	33
	hiPSCs	EB‐F	USP18 depletion enhances the responsiveness of PSCdM*ϕ*s to IFN‐*α*/*β*, thus curtails HIV replication within PSCdM*ϕ*s.	34
	hiPSCs	2D‐F	TLR3 activation enhances anti‐HIV activity of PSCdMGs by promoting the expression of IFN‐*α*/*β* and IFN‐*λ*.	35
IAV (HIV‐1, DENV)	hiPSCs	EB‐F	IRF5^−/−^ PSCdM*ϕ*s exhibited impaired IAV‐induced production of IL‐6 and TNF‐*α*.	36
	hiPSCs chimpanzee iPSCs	EB‐F	PSCdM*ϕ*s replicate HIV‐1, DENV and IAV similarly to human MDMs; PSCdM*ϕ*s derived from human and chimpanzee reveal differential susceptibility to DENV infection.	37
SARS‐CoV‐2	hESCs hiPSCs	2D‐F	Both M1 and M2 PSCdM*ϕ*s inhibits SARS‐CoV‐2 infection but lung cells undergo apoptosis or protections in co‐culture with M1 or M2 PSCdM*ϕ*s, respectively.	38
ASFV (PRRSV)	pEPSCs	EB‐F	PSCdM*ϕ*s are highly susceptible to ASFV and PRRSV infection and sustain these viral replication and production.	39
*Mycobacterium tuberculosis*	hESCs hiPSCs	2D‐F	High‐throughput screening and experimental validation identified 10DEBC as a novel compound active against *M. tuberculosis*.	40
	hiPSCs	EB‐S	IFNGR1 deficiency abolished IFN‐*γ*‐dependent BCG clearing.	41
	hiPSCs	EB‐S	Upon IFN‐*γ* stimulation or BCG infection, PSCdM*ϕ*s derived from patients with inborn errors in IFN‐*γ* immunity show similar deficiencies to those of clinical phenotypes.	42
	hiPSCs hESCs	EB‐S	In response to BCG infection, PSCdM*ϕ*s exhibited immunological function by apoptosis, elevated NO production and increased expression of TNF‐*α*.	43
	hiPSCs	EB‐S	PSCdM*ϕ*s are similar to pulmonary M*ϕ*s rather than MDMs; PSCdM*ϕ*s restrict the growth of *M. tuberculosis* by >75% higher than MDMs.	44
*Leishmania*	hiPSCs	EB‐S	In PSCdM*ϕ*s infected with *Leishmania*, the reference drug amphotericin B shows similar EC_50_ values to those of previous reports.	45
	hiPSCs	EB‐S	The IC_50_ values of reference drugs miltefosine and amphotericin B in PSCdM*ϕ*s infected with *Leishmania* are comparable with those in other M*ϕ* models.	46
*Chlamydia trachomatis*	hiPSCs	EB‐F	*C. trachomatis* evades lysosomal fusion in PSCdM*ϕ*s for complete infection lifecycle; IRF5 and IL10RA limit *C. trachomatis* infection in PSCdM*ϕ*s.	47
*Staphylococcus aureus*	hiPSCs	EB‐S	Deletion of PVL receptor, C5aR1, protects PSCdM*ϕ*s from the cytotoxicity by PVL, one of the leukocidins secreted by *S. aureus*.	48
*Pseudomonas aeruginosa*	hiPSCs	EB‐S	Bioreactor‐derived PSCdM*ϕ*s effectively rescue mice from *P. aeruginosa* infections in the lower respiratory tract after being transplanted into the lungs.	49
*Salmonella*	hiPSCs	EB‐S	PSCdM*ϕ*s efficiently phagocytize and restrict the growth of *S. typhi and S. typhimurium*.	24

Abbreviations: 10DEBC, 10–4′‐(N,N‐diethylamino)butyl‐2‐chlorophenoxazine hydrochloride; 2D‐F, embryoid body‐independent factor‐assisted (protocols); ASFV, African swine fever virus; BCG, Bacillus Calmette‐Guérin; CB, cord blood; CCR5, C‐C chemokine receptor type 5; CMP, common myeloid progenitor; CNS, central nervous system; CXCR4, C‐X‐C motif chemokine receptor 4; DENV, dengue virus; dpi, days post‐infection; EB‐F, embryoid body‐based factor‐assisted (protocols); EB‐S, embryoid body‐based spontaneous (protocols); EC_50_, concentration for 50% of maximal effect; hESCs, human embryonic stem cells; hiPSCs, human induced pluripotent stem cells; HIV‐1, human immunodeficiency virus type 1; HLA‐DR, human leukocyte antigen DR; IAV, influenza A virus; IC_50_, half‐maximal inhibitory concentration; IFNAR2, interferon alpha and beta receptor subunit 2; IFN‐*α*/*β*, interferons alpha/beta; IFN‐*λ*, interferons lambda; IFN‐*γ*, interferon‐gamma; IFNGR1, interferon‐gamma receptor 1; IL10RA, interleukin 10 receptor *α*; IL‐6, interleukin 6; IRF5, interferon regulatory factor 5; M1, proinflammatory macrophages; M2, anti‐inflammatory macrophages; MDMs, blood monocyte‐derived macrophages; MIF, migration inhibitory factor; M*ϕ*s, macrophages; NF‐*κ*B, nuclear factor‐kappa B; NHP, nonhuman primate; NLRP3, NLR family pyrin domain containing 3; NO, nitric oxide; OP9, bone marrow‐derived stromal cells; PBMCs, peripheral blood mononuclear cells; pHHs, primary human hepatocytes; PRRSV, porcine reproductive and respiratory syndrome virus; PSCdMGs, pluripotent stem cell‐derived microglia; PSCdM*ϕ*s, pluripotent stem cell‐derived macrophages; PVL, Panton–Valentine leucocidin; SARS‐CoV‐2, severe acute respiratory syndrome coronavirus 2; SIV, simian immunodeficiency viruses; TLR3, Toll‐like receptor 3; TNF‐*α*, tumour necrosis factor alpha; USP18, ubiquitin‐specific proteinase 18; ZIKV, Zika virus.

### 2.1 Protocols for the generation of PSCdM*ϕ*s

**FIGURE 1:**
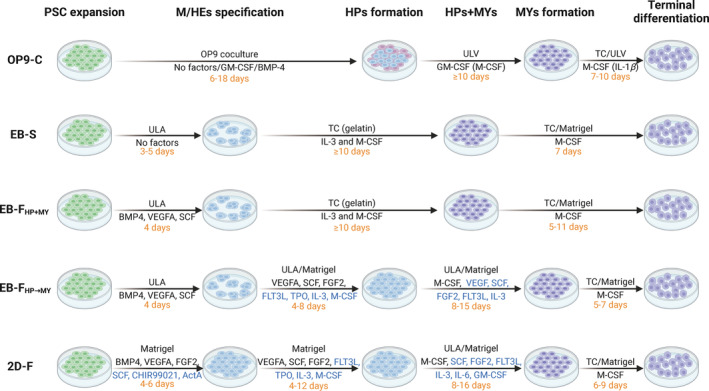
Schematic representation for PSC differentiation into PSC‐derived M*ϕ*s (PSCdM*ϕ*s) induced by different protocols. Four protocols for generation of PSCdM*ϕ*s are divided into four stages: the induction of mesoderm and haemogenic endothelia (M/HEs), the formation of haematopoietic progenitors (HPs), the production of monocyte‐like cells (MYs) and the terminal differentiation (TD) of MYs into PSCdM*ϕ*s. In the OP9‐C protocols, PSCs co‐cultured with OP9 cells are differentiated into HPs without exogenous factors or sometimes with GM‐CSF or BMP‐4 treatment. MYs are then induced upon GM‐CSF or M‐CSF treatment under ultra‐low adsorption (ULA) condition, followed by differentiation into PSCdM*ϕ*s in the presence of M‐CSF (or IL‐1*β*) in tissue culture (TC) plates or under ULA condition. In the EB‐S protocols, PSCs are spontaneously differentiated into M/HEs under ULA condition without exogenous factors. The M/HEs are then adherently cultured in gelatine‐coated TC plates with medium containing M‐CSF and IL‐3 to simultaneously induce the formation of HPs and MYs, which are then be differentiated into PSCdM*ϕ*s upon M‐CSF treatment in TC plates or Matrigel‐coated plates. The EB‐F_HP+MY_ protocols are the modified version of the EB‐S protocols, where several exogenous factors including BMP‐4, VEGFA and SCF are used to enhance the differentiation efficiency of M/HEs. Modified from the EB‐F_HP+MY_ protocols, the EB‐F_HP→MY_ protocols use different combinations of exogenous factors to continuously induce HPs and MYs formation, and finally generation of PSCdM*ϕ*s under M‐CSF treatment. In the 2D‐F protocols, different combinations of exogenous factors induced the formation of each stage in Matrigel‐coated plates and finally the generation of PSCdM*ϕ*s. ActA, activin A; BMP4, bone morphogenetic protein 4; CHIR99021, GSK inhibitor/Wnt activator; FGF2, basic fibroblast growth factor; FLT3L, FMS‐like tyrosine kinase 3 ligand; GM‐CSF, granulocyte‐macrophage colony‐stimulating factor; IL‐3, interleukin 3; IL‐6, interleukin 6; M‐CSF, macrophage colony‐stimulating factor; SCF, stem cell factor; TPO, thrombopoietin; VEGFA, vascular endothelial growth factor A. Blue font for different combinations of exogenous factor. Created with BioRender.com.

Different strategies for generating PSCdM*ϕ*s have been discussed in several reviews.^18^, ^19^, ^23^, ^50^, ^51^ Generally, the generation of PSCdM*ϕ*s consists of four main steps^50^: (i) mesoderm commitment and specification of haemogenic endothelia (M/HEs); (ii) endothelial‐to‐haematopoietic transition and the generation of haematopoietic progenitors (HPs); (iii) myeloid specification and the formation of monocyte‐like cells (MYs); and (iv) terminal differentiation (TD) of MYs into PSCdM*ϕ*s. Based on the M/HEs stage, different protocols have been categorized into four types (Figure 1): (i) OP9 (bone marrow‐derived stromal cells)‐co‐culture (OP9‐C) protocols; (ii) embryoid body (EB)‐based spontaneous (EB‐S) protocols; (iii) EB‐based factor‐assisted (EB‐F) protocols; and (iv) EB‐independent factor‐assisted (2D‐F) protocols.^18^

The OP9‐C protocols were the first to obtain PSCdM*ϕ*s, by which ESCs or iPSCs were co‐cultured with OP9 cells to generate haematopoietic progenitors, followed by differentiation into PSCdM*ϕ*s using macrophage colony‐stimulating factor (M‐CSF) or granulocyte‐macrophage colony‐stimulating factor (GM‐CSF).^52^, ^53^, ^54^ In the EB‐S protocols, EBs are spontaneously or artificially formed, facilitating the communication of PSCs and progression to the M/HEs stage.^18^ The M/HEs stage is subjected to treatment with M‐CSF plus IL‐3, and sequentially M‐CSF alone, to harvest MYs and PSCdM*ϕ*s, successively.^44^, ^55^, ^56^, ^57^ In the EB‐based factor (EB‐F)‐assisted protocols, multiple exogenous factors contribute to M/HEs formation and the generation efficiency of PSCdM*ϕ*s. These exogenous factors include vascular endothelial growth factor A (VEGFA), stem cell factor (SCF), fibroblast growth factor 2 (FGF2), FMS‐like tyrosine kinase 3 ligand (FLT3L) and thrombopoietin (TPO).^58^ In the 2D‐F protocols, PSCs are cultured adherently with Matrigel, restricting the three‐dimensional diffusion of PSCs.^59^, ^60^ Consequently, exogenous factors, including BMP‐4, VEGF, SCF, FGF2, CHIR99021 and activin A, are used to drive the formation of the HPs and MYs, thus improving the induction efficiency of PSCdM*ϕ*s.

### 2.2 Pathogen–PSCdM*ϕ* interactions

**FIGURE 2:**
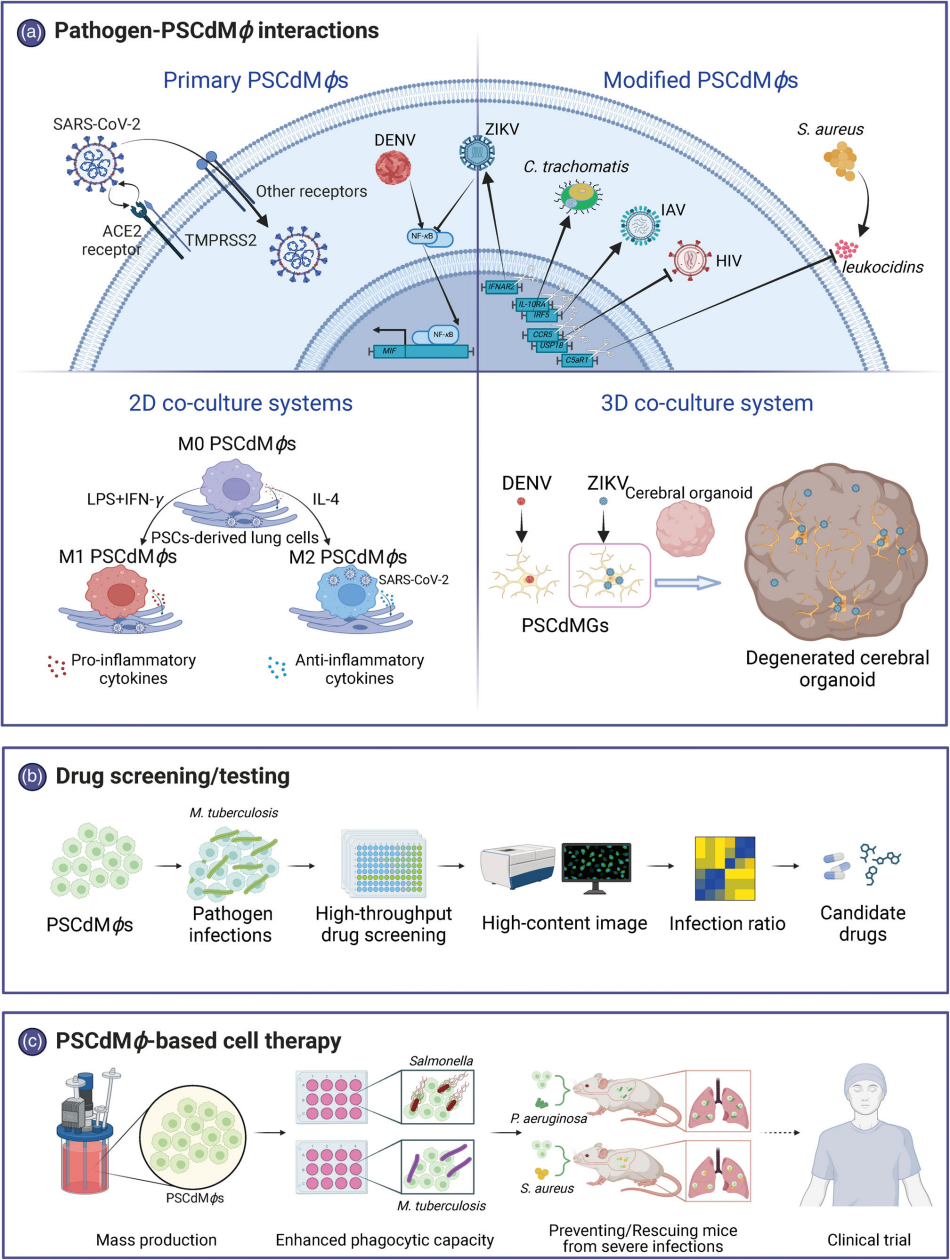
Schematic representation for the implications of PSCdM*ϕ*s in pathogenesis studies. (A) PSCdM*ϕ*s serve as efficient models for dissecting pathogenesis of various pathogens, and both 2D and 3D co‐culture system confer better physiological relevance to PSCdM*ϕ*s. (B) The flow chart of drug test or screening based on high‐content screening platform. (C) In vitro evaluation of bactericidal ability and *in vivo* PSCdM*ϕ*‐based cell therapy assays for PSCdM*ϕ*s. ACE2, angiotensin‐converting enzyme 2; C5aR1, complement 5a receptor 1; CCR5, CC chemokine receptor 5; DENV, dengue virus; HCV, hepatitis C virus; HIV, human immunodeficiency virus; IAV, influenza A virus; IFNAR2, interferon alpha and beta receptor subunit 2; IFN‐*γ*, interferon‐gamma; IL‐10RA, interleukin 10 receptor subunit alpha; IL‐4, interleukin 4; IRF5, interferon regulatory factor 5; LPS, lipopolysaccharide; M0, nonactivated macrophages; M1, proinflammatory macrophages; M2, anti‐inflammatory macrophages; MIF, macrophage migration inhibitory factor; NF‐*κ*B, nuclear factor‐kappa B; PSCdMGs, pluripotent stem cell‐derived microglia; SARS‐CoV‐2, severe acute respiratory syndrome coronavirus 2; TMPRSS2, transmembrane serine protease 2; USP18, ubiquitin‐specific proteinase 18; ZIKV, Zika virus. Created with BioRender.com.

An important topic in studying infectious diseases is to analyze the pathogen–PSCdM*ϕ* interactions, where PSCdM*ϕ*s have become the important tools (Figure 2A). PSCdM*ϕ*s efficiently support the infection and replication of human immunodeficiency virus (HIV) and dengue virus (DENV), while inhibiting replication during the late stages of the IAV life cycle.^37^ PSCdM*ϕ*s have also been utilized to delineate distinct immune responses to Zika virus (ZIKV) and DENV infections. Specifically, DENV infection is associated with increased secretion of macrophage migration inhibitory factor (MIF) and reduced cell migration. Conversely, ZIKV infection disrupts the nuclear factor‐kappa B (NF‐*κ*B)‐MIF feedback loop and leads to the inhibition of the NF‐*κ*B signalling, representing a key mechanistic difference of these two viruses.^25^ The PSC‐derived microglia (PSCdMGs) exhibit mild cytotoxicity when infected with ZIKV but not DENV, in contrast to the higher cytotoxicity of other cell types of central nervous system.^26^

The compatibility of PSCs with gene editing technologies, such as transcription activator‐like effector nucleases (TALENs) and clustered regularly interspaced short palindromic repeats (CRISPR)‐associated nuclease 9 (CRISPR/Cas9) genome editing system, facilitates the studies identifying host factors in infectious diseases, including the infection of HIV, IAV and ZIKV.^27^, ^34^, ^36^, ^48^ Among these, HIV is one of the most studied viruses. In multiple studies, *CCR5*, a key coreceptor required for M*ϕ*‐trophic strains of HIV, was disrupted or seamlessly modified using the piggyBac technology plus TALENs or CRISPR/Cas9 system, conferring PSCdM*ϕ*s resistance to HIV,^28^, ^29^, ^30^, ^31^ demonstrating the potential of *CCR5* as a therapeutic target. Additionally, *C. trachomatis* evades lysosomal fusion within PSCdM*ϕ*s to form mature inclusions and ultimately highly infectious progeny are released, demonstrating the ability of *C. trachomatis* to utilize M*ϕ*s as dissemination vectors.^61^, ^62^, ^63^, ^64^ Furthermore, knockouts of interferon regulatory factor 5 (IRF5) and IL‐10 receptor subunit alpha (IL‐10RA) by CRISPR/Cas9 demonstrated their roles in limiting *C. trachomatis* in PSCdM*ϕ*s.^47^

Animal defence against pathogens is a holistic activity involving the coordinated work of different tissues and different cells. While studying the pathogen‐PSCdM*ϕ* interactions based on a single‐cell type is feasible, co‐culture systems can better reproduce the realistic biological process. Nonactivated M*ϕ*s (M0) can either be classically activated as proinflammatory M*ϕ*s (M1) by LPS plus IFN‐*γ* or be alternatively activated as anti‐inflammatory M*ϕ*s (M2).^65^, ^66^ M1 and M2 have been reported to promote or inhibit, respectively, the spread and containment of severe acute respiratory syndrome coronavirus 2 (SARS‐CoV‐2), which contrasts with HIV and SIV.^67^, ^68^, ^69^ However, in co‐culture with lung cells, both M1 and M2 PSCdM*ϕ*s inhibit SARS‐CoV‐2 infections, albeit in distinct manners.^38^ Specifically, M1 PSCdM*ϕ*s up‐regulate inflammatory factors to eliminate SARS‐CoV‐2, but also suppress growth and induce apoptosis of lung cells, resulting in further pulmonary inflammation and damage. In contrast, M2 PSCdM*ϕ*s exhibit enhanced phagocytic activity and anti‐inflammatory responses upon SARS‐CoV‐2 infection, providing efficient protection for lung cells and PSCdM*ϕ*s. Additionally, when co‐cultured with brain organoids, PSCdMGs infected with ZIKV penetrated the neural tissue and resulted in their degeneration,^26^ suggesting that microglia (MGs) may act as viral reservoirs facilitating the establishment of ZIKV infection within the foetal brain, thus leading to vertical transmission of ZIKV from mother to foetus. Overall, the co‐culture system preserves the cell‐to‐cell interactions and accurately reflects the pathogen–PSCdM*ϕ* interactions.

### 2.3 Drug screening and testing

Currently, the M*ϕ* models commonly used for drug screening and testing include THP‐1, RAW264.7 and BMDMs. Indeed, studies have reported the use of PSCdM*ϕ*s to test and screen for drugs that significantly affect human health. Given the insufficiency of nontransformed M*ϕ* models for anti‐tuberculosis drug screening, a modified method was established to generate homogeneous PSCdM*ϕ*s susceptible to infection by *M. tuberculosis*, producing about 25 million PSCdM*ϕ*s from each input colony of human ESCs (hESCs) or human iPSCs (hiPSCs).^40^ This approach enabled large‐scale drug screening against *M. tuberculosis*, and after further validation in a secondary screen, 10‐DEBC, one of the 120 hits, was shown to exert activity against drug‐resistant strains (Figure 2B). Moreover, PSCdM*ϕ*s exhibited relative susceptibility to multiple *Leishmania strains*, which was inhibited by two reference drugs, suggesting that PSCdM*ϕ*s represent an effective M*ϕ* model for screening and testing drugs against infectious diseases.^45^, ^46^ iPSC technology facilitates the application of precision medicine, enabling the evaluation of patient‐specific drugs and the development of effective therapies. Mendelian susceptibility to mycobacterial diseases is a congenital disease characterized by selective susceptibility to infections by intra‐macrophagic pathogens, including *M. tuberculosis*, predominantly attributed to congenital errors in IFN‐*γ* immunity.^70^ Patient‐specific PSCdM*ϕ*s with autosomal recessive disruptions of interferon‐gamma receptor 1 (IFNGR1), interferon‐gamma receptor 1 (IFNGR2) or STAT1 were generated from individuals and showed impaired IFN‐*γ* signalling and functionality when stimulated with IFN‐*γ* or challenged with the Bacillus Calmette–Guérin vaccine.^41^, ^42^ These studies underscore the potential of PSCdM*ϕ*s in assessing novel therapeutic approaches and investigating mycobacterial infections.

### 2.4 PSCdM*ϕ* ‐based cell therapy

In addition to serving as a model, PSCdM*ϕ*s also demonstrate enormous potential in cell therapy for infectious diseases. The resilience and adaptability of some pathogens, stemming from their extensive genomes containing a large array of regulatory genes and virulence factors, along with their ability to resist antimicrobial agents, categorize them as a formidable bacterial adversary.^71^ Therefore, while antibiotics remain the primary treatment for bacterial infections, the rise of antibiotic‐resistant variants and the crucial functions of M*ϕ*s in lung immunity suggest that PSCdM*ϕ*‐based cell therapy could serve as an effective alternative strategy for managing severe bacterial infections of the respiratory system (Figure 2C).

PSCdM*ϕ*s produce pronounced cytokines and effectively eradicate *Salmonella typhi* and *S. typhimurium*,^24^ demonstrating their potentials in cell therapy against bacterial infections. Ackermann innovatively introduced the PSCdM*ϕ*‐based cell therapy that were compatible with mass‐production in stirred‐tank bioreactors, which prevented the infections of *Pseudomonas aeruginosa* and rescued mice from severe infections, showcasing the potential of PSCdM*ϕ*‐based cell therapy for bacterial infections.^49^, ^72^ Additionally, low‐activated/low‐polarized ‘naïve‐like’ PSCdM*ϕ*s were established and effectively inhibited *M. tuberculosis* growth in vitro.^44^ For drug‐resistant strains, PSCdM*ϕ*‐based cell therapy demonstrates greater clinical significance. The adoptive transfer of PSCdM*ϕ*s led to a significant reduction of the loads of *Staphylococcus aureus* resisting methicillin in murine airway, which was associated with diminished granulocyte infiltration and lessened pulmonary tissue damage in the treated mice, indicating an important characteristic for therapeutic use.^73^

## 3 SRM*ϕ*s: EXPANDABLE BUT NONTRANSFORMED M*Φ* MODELS

**FIGURE 3:**
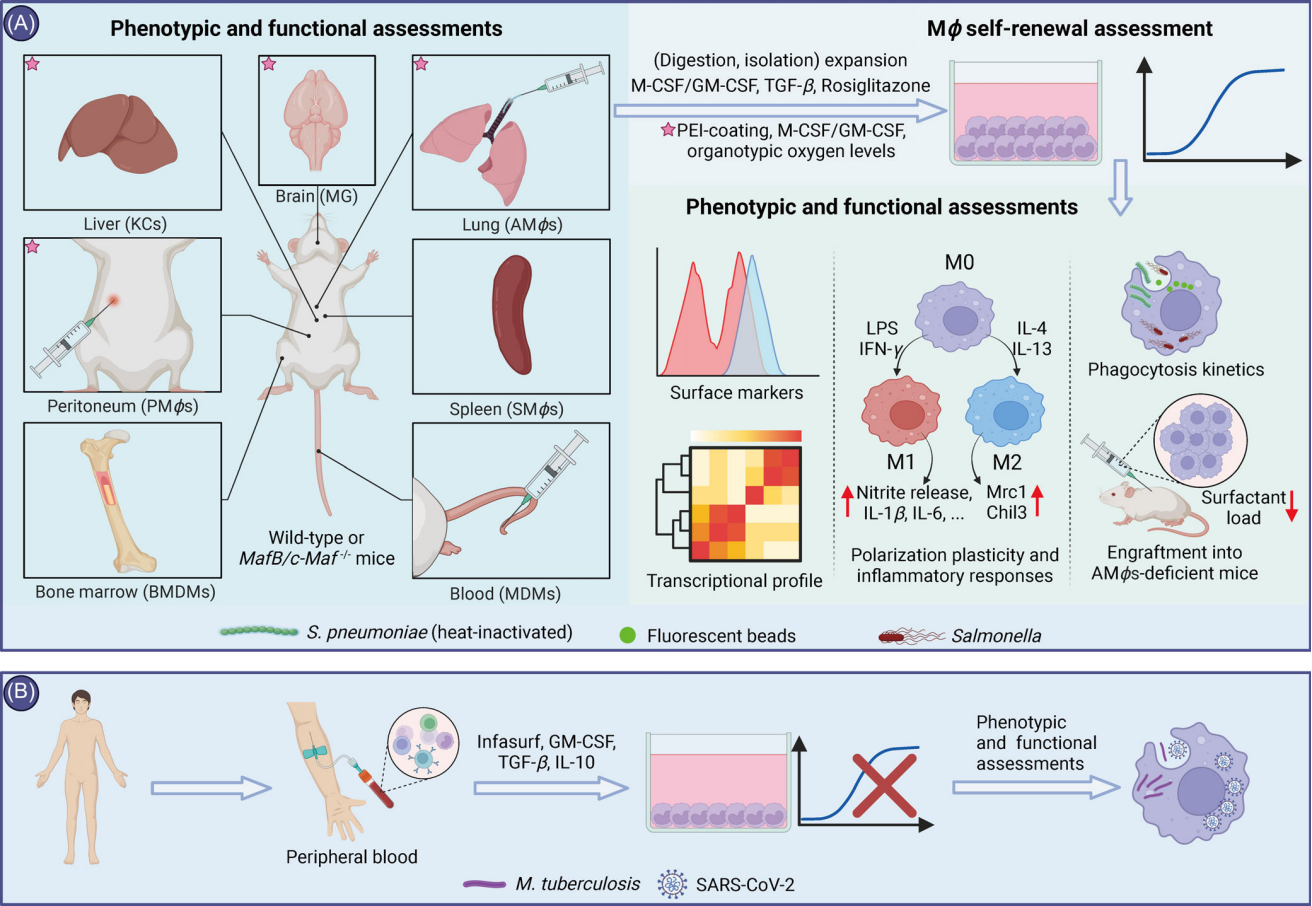
Acquisition and characterization of SRM*ϕ*s. (A) Multiple organ‐derived SRM*ϕ*s and the characterization of phenotypes and functions. Asterisks represent organs and culture conditions involved in improved organotypic M*ϕ* culture system. (B) Monocyte‐derived AM*ϕ*‐like cells from human peripheral blood and the phenotype and function assessments. AM*ϕ*s, alveolar macrophages; BMDMs, bone marrow‐derived macrophages; Chil3, chitinase 3‐like 3; GM‐CSF, granulocyte‐macrophage colony‐stimulating factor; IFN‐*γ*, interferon‐gamma; IL‐10, interleukin 10; IL‐13, interleukin 13; IL‐1*β*, interleukin 1 beta; IL‐4, interleukin 4; IL‐6, interleukin 6; KCs, Kupffer cells; LPS, lipopolysaccharide; M0, nonactivated macrophages; M1, proinflammatory macrophages; M2, anti‐inflammatory macrophages; M‐CSF, macrophage colony‐stimulating factor; MDMs, monocyte‐derived macrophages; MG, microglia; MRC1, mannose receptor C‐type 1; PM*ϕ*s, peritoneum macrophages; rosiglitazone, an agonist of peroxisome proliferator‐activated receptor gamma; SARS‐CoV‐2, severe acute respiratory syndrome coronavirus 2; SM*ϕ*s, spleen macrophages; TGF‐*β*, transforming growth factor beta. Created with BioRender.com.

Generally, highly differentiated cells lose the self‐renewal capacity characteristic of PSCs.^74^ Although PSCdM*ϕ*s have been extensively utilized in the modelling and cell therapy of infectious diseases, their challenges in proliferation necessitates large‐scale expansion of PSCs for substantial production of PSCdM*ϕ*s. This requirement poses challenges in terms of reducing both the cost and cycle time for the culture of PSCs and the differentiation of PSCdM*ϕ*s. Fortunately, studies have shown that SRM*ϕ*s can be generated through special treatment, including genetic manipulation, stimulation with growth factor cocktails and culture within organotypic system (Figure 3A,B). These previously overlooked approaches may provide a solution for the efficient production of PSRM*ϕ*s.

### 3.1 *MafB/c‐Maf*: the obstacle of establishing SRM*ϕ*s

The bZip transcription factors MafB and c‐Maf have been reported to respond against treatment of M‐CSF.^75^, ^76^ Aziz et al. were the first to discover that primary M*ϕ*s isolated from mice with simultaneous knockout of both *MafB* and *c‐Maf* genes can proliferate exponentially under M‐CSF stimulation.^20^ Similarly, naturally low MafB/c‐Maf levels drive self‐renewal in AM*ϕ*s.^77^ Specifically, *MafB/c‐Maf*‐DKO in M*ϕ*s releases the constraints on Ets‐1/2^78^ and PU.1,^76^, ^79^ activating the expression of *c‐Myc* and *Krüppel‐like factor 4* (*KLF4*), thereby sustaining the self‐renewal ability of M*ϕ*s, termed DKO‐M*ϕ*s. These DKO‐M*ϕ*s maintain a consistent phenotype and functionality without transformation, exhibiting sustained and stable proliferation for up to 8 months.^20^ Furthermore, analysis of enhancer repertoires using chromatin immunoprecipitation sequencing revealed a gene network that governs the self‐renewal of DKO‐M*ϕ*s. Interestingly, this gene network also controls the self‐renewal of ESCs, in spite of employing completely distinct enhancer repertoires.^22^ The enhancer repertoire specific for M*ϕ*s is negatively regulated by MafB and c‐Maf. Consequently, depleting MafB and c‐Maf could confer proliferative capabilities to M*ϕ*s, thus generating SRM*ϕ*s.^22^ These findings highlight the role of *MafB/c‐Maf* as the obstacle to establishing SRM*ϕ*s, challenge the dogma that terminal differentiation precludes the re‐entry into cell cycle and suggest a framework for scrutinizing self‐renewal mechanisms in other cell types.

Similar to the silent information regulator 2 (SIR2) in *Saccharomyces cerevisiae*,^80^, ^81^ the mammalian homologue sirtuin 1 (SIRT1) has been implicated in governing stem cell self‐renewal.^82^, ^83^, ^84^ Interestingly, SIRT1 has also been suggested to be a pivotal master regulator of M*ϕ*s self‐renewal, integrating terminal differentiation and proliferation.^21^ Specifically, this involves the expression of *E2F1* and *c‐Myc*, as well as the concurrent inhibition of *FOXO1* that regulates cell cycle progression.^21^ These findings further support the notion that SRM*ϕ*s and ESCs shared a gene network in charge of self‐renewal.

### 3.2 Growth factors: the proliferation clue to SRM*ϕ*s

Several growth factors, including colony‐stimulating factor, transforming growth factor beta (TGF‐*β*) and rosiglitazone, an agonist of peroxisome proliferator‐activated receptor gamma (PPAR‐*γ*), have been reported to endow M*ϕ*s with the ability of self‐renewal by mimicking the in vivo environment.^85^, ^86^, ^87^, ^88^ Given the modulation mechanism of M*ϕ* functions,^89^ recombinant GM‐CSF has been utilized to stimulate the primary isolated murine AM*ϕ*s, endowing them with the ability for sustained self‐renewal in vitro.^86^ Given the development of M*ϕ*s, foetal liver was used to establish SRM*ϕ*s, designated as Max Planck Institute (MPI) cells, which proliferate in a GM‐CSF/STAT5‐dependent manner without transformation.^90^ These MPI cells manifest the innate immune characteristics of AM*ϕ*s and maintain their phenotypes and functions for up to 2 years in a stable state.^90^ Intriguingly, the expression levels of *MafB* and *c‐Maf* were observed to be relatively low in MPI cells, whereas *KLF4* and *c‐Myc* displayed heightened expression levels, further elucidating the regulatory mechanisms underlying M*ϕ* proliferation. In addition to embryonic progenitors, circulating monocytes can also develop into SRM*ϕ*s.^91^, ^92^, ^93^ In a mouse model depleting KCs, circulating monocytes underwent differentiation and engrafted in the liver, displaying a transcriptional pattern akin to that of KCs.^93^ M‐CSF‐dependent SRM*ϕ*s were established by repetitively detaching and reseeding BMDMs. The underlying mechanism driving self‐renewal involves the downregulation expression of *MafB* and the upregulation of *Krüppel‐like factor 2* (*KLF2*), reminiscent of *KLF4*.^94^ Hence, like embryonic precursor cells, circulating monocytes have the potential to give rise to SRM*ϕ*s.

Distinct from other tissue‐resident M*ϕ*s (TRMs), the development of AM*ϕ*s requires not only GM‐CSF but also self‐secreted TGF‐*β*, leading to subsequent upregulation release of PPAR‐*γ*. Together, these growth factors together contribute to the growth and division of AM*ϕ*s.^95^, ^96^ To establish SRM*ϕ*s physiologically similar to AM*ϕ*s, GM‐CSF, TGF‐*β* and rosiglitazone were used to emulate the in vivo microenvironment of AM*ϕ*s.^85^, ^87^, ^88^ By this approach, monocytes from bone marrow and foetal liver of mice were differentiated into AM*ϕ*‐like (AML) cells in vitro, whose phenotypes and functions were similar to those of primary AM*ϕ*s.^85^, ^88^ Remarkably, these growth factors also promoted the proliferation of bronchoalveolar lavage fluid (BALF)‐derived AM*ϕ*s in vitro, expanding the M*ϕ* population by over 20‐fold after 9 days of culture.^85^, ^88^ Similarly, postnatal mouse hepatic cells were differentiated into AML cells, which expanded for several months while preserving their typical phenotypes and functions, effectively ameliorating the symptoms of pulmonary alveolar proteinosis in AM*ϕ*‐deficient mice.^87^ Additionally, natural bovine surfactant Infasurf and interleukin 10 (IL‐10), were included in the differentiation of human circulating monocytes into the AML cells (Figure 3B). These AML cells exhibited susceptibility akin to human AM*ϕ*s towards both *M. tuberculosis* and SARS‐CoV‐2.^97^ However, despite obtaining 12.5 to 25 times more AML cells per donor from whole blood compared with BALF, these AML cells exhibit a limited proliferation potential.^97^ These findings may indicate the interspecies differences in the in vivo microenvironment of AM*ϕ*s, necessitating further research.

### 3.3 Organotypic culture system: a more authentic niche for SRM*ϕ*s

Although growth factor cocktails promote M*ϕ* proliferation, they inevitably differ from their in vivo counterparts, which may be addressed by more sophisticated culture systems. Recently, an organotypic cell culture system has been established to sustain TRMs for several weeks without the need for purification, including KCs, AM*ϕ*s, MGs and peritoneum macrophages (PM*ϕ*s).^98^ To better simulate the in vivo microenvironment and enhance cell production, the system adopted three measures: (1) culturing TRMs in polyethylenimine‐coated plates to remove contaminants and maintain TRM purity for 14 days; (2) supplementing the culture medium with specific growth factors (M‐CSF, GM‐CSF and IL‐34) and refreshing 40% to retain trophic factors; and (3) using organotypic oxygen levels, with AM*ϕ*s at 3% oxygen and others at 22% oxygen. By this organotypic culture system, TRMs maintained core M*ϕ* surface markers and transcriptomic profiles for 14 days.^98^ Importantly, different TRMs exhibited high heterogeneity in gene expression in response to immunogenic stimuli, suggesting that TRMs may serve as a more ideal model for dissecting TRM behaviour than BMDMs.

Although still in its early stages, the organotypic culture system has optimized conditions to further simulate the microenvironment. With further development, *MafB/c‐Maf* ^−/−^ TRMs might be refined into SRM*ϕ*s that closely mimic in vivo M*ϕ*s. However, additional research is required to validate this potential.

### 3.4 Potential applications of SRM*ϕ*s

Notably, the applications of SRM*ϕ*s in other fields remain limited, which may be caused by several factors. Firstly, establishing SRM*ϕ*s faces challenges in genetic modification. Myeloid cells, including M*ϕ*s, are considered challenging to genetic manipulated due to resistance to exogenous nucleic acids and lentiviral vectors.^12^, ^13^ Improved gene editing technologies, such as the nucleofection‐based delivery of the Cas9‐ribonucleic proteins, show promise but require further validation for reliability.^99^ Secondly, the clinical applications for SRM*ϕ*‐based cell therapy require rigorous safety evaluation. Short‐term tumorigenesis studies suggest SRM*ϕ*s are safe,^20^ but their long‐term safety needs thorough evaluation to ensure their suitability for cell therapy. Interestingly, although fluctuations in gene expression of SRM*ϕ*s have been observed, their genetic expression patterns and epigenetic features resemble those of their in vivo counterparts for at least 4 months post‐transplantation, signifying the potential safe transplantation of expanded M*ϕ*s in vivo.^100^ Thirdly, the functionality and pathogen susceptibility of SRM*ϕ*s need detailed study, crucial for developing live attenuated vaccines (LAVs) for infectious diseases, such as African swine fever, which causes significant economic losses.^101^ Overall, comprehensive studies are needed to explore the feasibility of obtaining SRM*ϕ*s in other species and to rationally assess the safety and expand the applications of SRM*ϕ*s. A comparative analysis is performed to discuss the advantages and limitations among primary M*ϕ*s, M*ϕ* lines, PSCdM*ϕ*s and SRM*ϕ*s in modelling and treating infectious diseases (Table 2).

**TABLE 2:** The advantages and limitations of different M*ϕ* models in modelling and treating infectious diseases.

Parameter	Availability, quantities	Standardizability	Genetic manipulation	Production cycle	Cost	Safety	Applications	References
Primary M*ϕ*s	Limited	Poor standardizability	Limited by cell low proliferative activity and resistance to exogenous DNA	Origin‐dependent (AM*ϕ*s, 1 day; MDMs, 7 days)	Medium	High	Pathogen–M*ϕ* interactions Drug screening …	8, 9, 12, 13
M*ϕ* lines	Almost unlimited	Standardizable	Limited by resistance to exogenous DNA	About 2–3 days starting from cell thawing	Low	Low	Pathogen–M*ϕ* interactions Drug screening …	10, 11, 12, 13
PSCdM*ϕ*s	Almost unlimited at the level of PSCs	Standardizable	Limited by cell low proliferative activity and resistance to exogenous DNA, but feasible at the level of PSCs	At least 15–20 days starting from PSCs expansion	High	Requires examination	Pathogen–PSCdM*ϕ* interactions Drug screening PSCdM*ϕ*‐based cell therapy …	18, 23, 49, 50, 102
SM*ϕ*s	Almost unlimited	Standardizable	Requires examination	About 4 days starting from cell passage	Low	Requires examination	Requires examination	20, 22, 90, 100

Abbreviations: AM*ϕ*s, alveolar macrophages; M*ϕ*s, macrophages; MDMs, blood monocyte‐derived macrophages; PSCdM*ϕ*s, pluripotent stem cell‐derived macrophages; PSCs, pluripotent stem cells; SM*ϕ*s, self‐renewing macrophages.

## 4 PSRM*ϕ*s: ENVISIONING A PROMISING AVENUE FOR INVESTIGATING INFECTIOUS DISEASES AND THE CHALLENGES TO BE ADDRESSED

**FIGURE 4:**
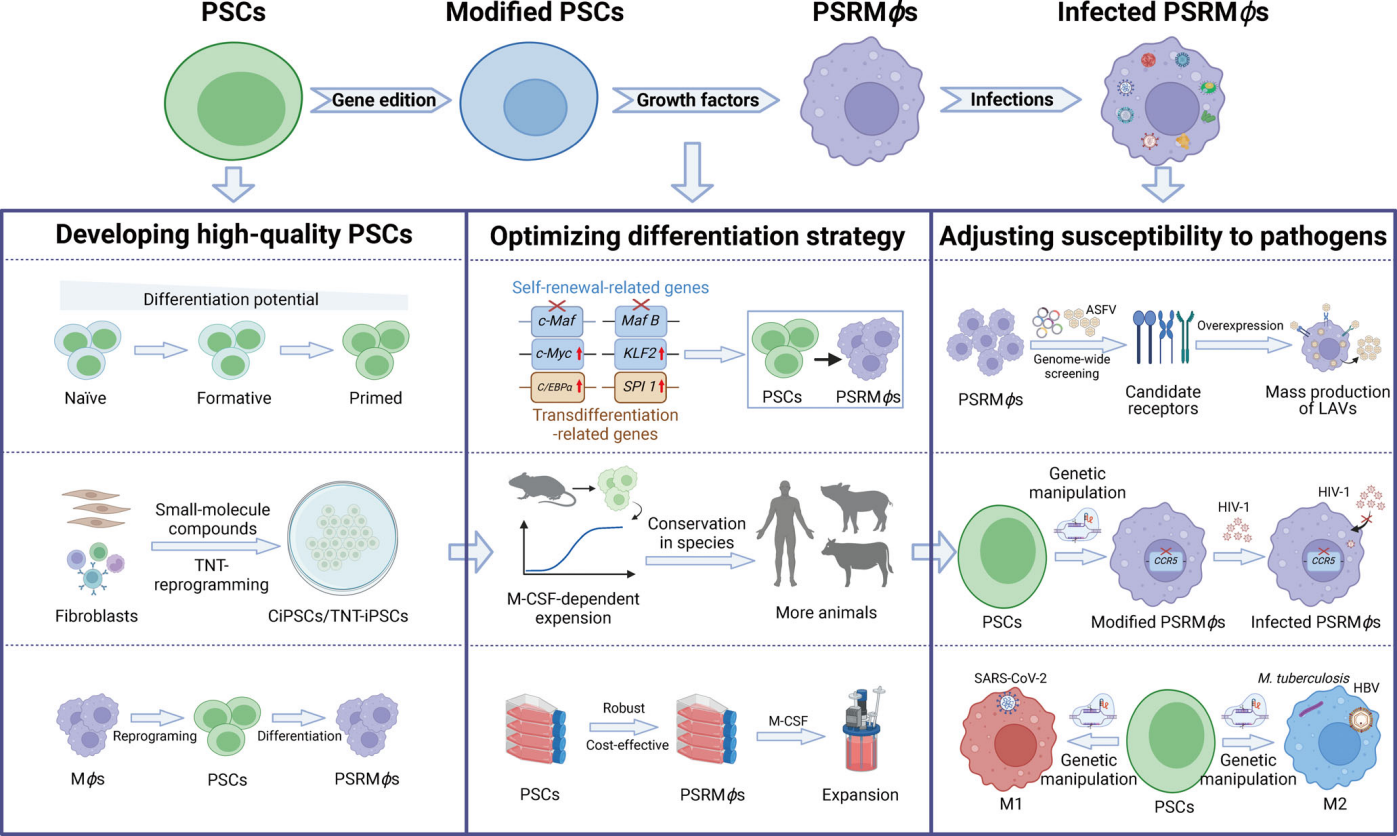
Envisioning powerful M*ϕ* models and the challenges to be addressed. Developing high‐quality PSCs is the prerequisite for developing PSC‐derived SRM*ϕ*s (PSRM*ϕ*s) with physiological relevance. Epigenetic memory modulates the differentiation potential of different PSCs. M*ϕ*‐derived iPSCs could provide an alternative source to create PSRM*ϕ*s. Optimizing differentiation strategy is the determinant for developing PSRM*ϕ*s. Considerations include the effects of self‐renewal‐related gene modifications on the pluripotency of PSCs, the universality of the M‐CSF‐dependent proliferation mechanisms of SRM*ϕ*s among multiple species, and the balance between standardization, robustness and cost‐effectiveness in the differentiation process. Adjusting susceptibility to pathogens as needed is crucial for applications of PSRM*ϕ*s. Improving the efficiency of ASFV replication in PSRM*ϕ*s will contribute to the mass production of live attenuated vaccines (LAVs). Knockout or mutation of CCR5, the coreceptor for HIV entry, would confer PSRM*ϕ*s non‐susceptibility to HIV infection. Generation of M1 PSRM*ϕ*s or M2 PSRM*ϕ*s by direct genetic modifications of PSCs allows rapid induction of pathogen‐specific susceptible polarized subtypes. ASFV, African swine fever virus; CiPSCs, chemically induced pluripotent stem cells; GM‐CSF, granulocyte‐macrophage colony‐stimulating factor; LAVs, live attenuated vaccines; M‐CSF, macrophage colony‐stimulating factor; PSCs, pluripotent stem cells; iPSCs, induced pluripotent stem cells; PSRM*ϕ*s, PSC‐derived self‐renewing macrophages; SARS‐CoV‐2, severe acute respiratory syndrome coronavirus 2; TNT, transient näive treatment. Created with BioRender.com.

Here, we propose that PSCs could serve as an effective platform for gene editing in PSCdM*ϕ*s, by targeting and disabling genes that hinder M*ϕ* self‐renewal within PSCs. These PSCs can then be differentiated into M‐CSF‐dependent SRM*ϕ*s, termed PSRM*ϕ*s, which is different from the malignant iPSC‐derived monocytic cells,^103^, ^104^ preserving the original functionality of M*ϕ*s and offering higher safety. Additionally, the gene editing on PSCs allows for diverse modifications of PSRM*ϕ*s, offering a valuable tool for M*ϕ* applications. Here, we discuss the challenges and potential solutions in the main stages of differentiating PSCs into PSRM*ϕ*s (Figure 4).

### 4.1 Prerequisite: developing high‐quality PSCs

The initial stage to acquire PSRM*ϕ*s is to obtain high‐quality PSCs. Although PSCdM*ϕ*s exhibit normal phenotypes and functions, they are slightly different from primary M*ϕ*s,^39^, ^105^ suggesting that developing high‐quality PSCs is the necessary prerequisite for creating PSCdM*ϕ*s with high physiological relevance.

The pluripotent state of PSCs is an important evaluation indicator of the quality of PSCs, which displays at least three forms: naïve‐, formative‐ and primed‐pluripotency.^106^, ^107^ In fact, naïve PSCs show several advantages over other PSCs. For example, the ability to passage and manipulate mouse ESCs (mESCs) at the single‐cell level allows for effective and precise genetic modifications.^108^ Additionally, under näive culture, the viability of hiPSCs and hESCs as well as their potential to be differentiated into mesoderm and endoderm are significantly improved.^109^, ^110^ Like mESCs, hESCs are isolated and cultured from preimplantation stage blastocysts.^111^ However, hESCs typically exhibit a primed‐ instead of naïve pluripotent state.^112^ While some studies suggest that this primed state can be converted to a naïve pluripotent state through genetic modifications or optimized culture systems,^113^, ^114^ more in‐depth research is needed.

In fact, epigenetic memory is a significant factor that affects the differentiation potential of PSCs.^115^, ^116^ Unlike ESCs, iPSCs retain DNA methylation signatures of the donor somatic cell, which limits the alternative cell fates of iPSCs.^115^, ^117^, ^118^ Several strategies have been successfully utilized to simulate the epigenetic landscapes of ESCs in iPSCs. The chemically induced pluripotent stem cells (CiPSCs) created with small molecular compounds exhibit transcriptomic profiles and epigenetic landscapes close to those of ESCs.^119^, ^120^, ^121^, ^122^ The transient‐näive‐treatment (TNT)‐reprogrammed iPSCs (TNT‐iPSCs) mimic the embryonic epigenetic reset and show higher efficiency than primed iPSCs in differentiation into skeletal muscle cells, which, like M*ϕ*s, are derived from the mesoderm.^123^ To date, the impacts of the epigenome of somatic cells on the functionality of PSCdM*ϕ*s have not been reported, and no research has been conducted comparing the differentiation of CiPSCs and TNT‐iPSCs into PSCdM*ϕ*s, which could greatly optimize the protocols for PSRM*ϕ* generation.

Dialectically examining the differentiation potential of iPSCs is beneficial for PSRM*ϕ* differentiation. As mentioned above, iPSCs retain DNA methylation signatures of the donor somatic cell, implying that iPSCs are more inclined to be differentiated into lineages related to the donor cells.^115^, ^117^, ^118^ Therefore, establishing iPSC lines reprogramming from M*ϕ*s, rather than the commonly used fibroblasts, may improve the PSRM*ϕ* differentiation, whose feasibility may be demonstrated by the successful establishment of iPSCs from peripheral blood mononuclear cells.^30^, ^124^ Therefore, M*ϕ*‐derived iPSCs may provide an alternative source for the establishment of PSRM*ϕ*s.

### 4.2 Linchpin: optimizing differentiation strategies

The second factor is the differentiation process of PSCs, regulated by core transcription factors, such as the octamer‐binding transcription factor 4 (Oct‐4), the sex‐determining region Y‐box 2 (Sox‐2) and the homeobox protein Nanog,^125^, ^126^ which work together to control genes maintaining pluripotency and guiding differentiation.^127^ In theory, it is feasible to remove genes that do not affect pluripotency in PSCs before differentiating them into target cells.^128^, ^129^, ^130^ However, it is currently unknown whether knocking out genes such as *MafB/c‐Maf*, which suppress M*ϕ* self‐renewal, impacts the differentiation of PSCs into M*ϕ*s or whether the principles for creating PSRM*ϕ*s have broad species applicability. This may be the linchpin in the creation of PSRM*ϕ*s.

In addition, employing an appropriate differentiation protocol will be crucial to balance the reproducibility, scalability, labour intensity, clinical relevance and costs of producing PSRM*ϕ*s.^18^, ^131^ The EB‐F protocols are the most balanced, offering high reproducibility with factor‐mediated control of the M/HEs stage, clinical applicability with xeno‐free conditions and cost‐efficiency using only IL‐3 and M‐CSF in HPs/MYs stages.^18^, ^39^, ^58^, ^59^ The EB‐S protocols, while cost‐effective and scalable, suffer from reduced reproducibility due to reliance on feeders and serum.^18^, ^44^, ^56^ The advantages of the 2D‐F protocols include xeno‐free conditions, a defined medium and factor‐dependent control over all differentiation stages, but these are offset by the necessity of using multiple factors and a single collection point, resulting in lower overall cell yield and increased costs.^18^, ^105^ While bioreactors have been employed to increase cell yield,^72^ this simultaneously raises the cost of differentiation. To enhance the stability, reproducibility and scalability of generating PSRM*ϕ*s, it may be promising to develop functionally closed and automated manufacturing platforms,^132^, ^133^ which automate most processing steps, reducing manpower demands and manufacturing deviations. Ideally, these automated manufacturing platforms also enable efficient utilization of cleanroom space, and incorporating in process analytics to monitor will promote the stable and reproducible generation of high‐quality PSRM*ϕ*s. Additionally, multiple quality control assays are essential during generation of PSRM*ϕ*s, which encompass determination of cell identity, cell viability, cytokine secretion, sterility testing and mycoplasma examination.

Moreover, the duration of the differentiation process is another factor to consider.^18^ Overexpression of lineage‐specific transcription factors in PSCs may be an effective strategy to improve the differentiation and simultaneously shorten the PSRM*ϕ* manufacturing. The enforced expression of *CCAAT/enhancer binding protein alpha* (*C/EBPα*) *and SPI1* rapidly induce the transdifferentiation of M*ϕ*s from T cells, B cells and fibroblasts within 5 days.^134^, ^135^, ^136^ These findings underscore the critical roles of C/EBP*α* and PU.1 (encoded by *SPI1*) in M*ϕ* differentiation and imply that their overexpression in PSCs may not only promote the differentiation of PSRM*ϕ*s but also shorten their manufacturing. Additionally, most differentiation protocols are the improvements of previously reported methods, with no studies reporting the systematic optimization of optimal growth factor combinations or differentiation durations using advanced techniques such as high‐throughput screening (HTS). Moreover, the HTS of chemical compounds could potentially enhance differentiation of PSRM*ϕ*s while reducing the costs and shorten the manufacturing.

### 4.3 Modification: adjusting susceptibility to pathogens

Due to the varying responses of M*ϕ*s in diverse infectious diseases, it is necessary to engineer M*ϕ*s as needed to modulate their susceptibility to and replication efficiency of pathogens. When studying certain pathogens devoid of a susceptible and faithful M*ϕ* model, PSRM*ϕ*s must accurately reflect M*ϕ* responses to pathogens for elucidating pathogen‐PSCdM*ϕ* interactions or conducting drug screening. Additionally, when PSRM*ϕ*s are intended for cell therapy to treat infectious diseases, it is crucial to enhance their pathogen clearance capabilities. Taking ASF as an example, the vaccination with LAVs remains a potential strategy for the prevention and control of ASF outbreaks in the future.^101^, ^137^ However, existing M*ϕ* models, including PSCdM*ϕ*s, exhibit insufficient susceptibility to ASFV compared with primary M*ϕ*s,^138^, ^139^, ^140^, ^141^ suggesting that PSRM*ϕ*s may encounter similar issues. Therefore, engineering PSRM*ϕ*s to support mass productions of ASFV LAVs is necessary, and various methods may help achieve this, among which overexpression of ASFV entry receptors by genetic manipulation at PSC levels may be the simplest and most effective way. However, the key entry receptors of ASFV have not been identified, which is hindered partly due to the insufficiency of expandable and nontransformed M*ϕ* lines.^140^ Fortunately, the compatibility of PSRM*ϕ*s with CRISPR libraries may contribute to finding the key entry receptors of ASFV. In conclusion, PSRM*ϕ*s may play important roles in versatile aspects of ASFV research.

Additionally, the susceptibility to pathogens can be modulated by altering various biological characteristics of PSRM*ϕ*s. Numerous M*ϕ* factors have been identified to be associated with susceptibility to pathogens, such as polarization states,^142^, ^143^ cytoskeleton^144^ and cell communication.^145^ Taking the polarization states of M*ϕ*s as an example, different pathogens exhibit preferences for the inflammatory response states of M*ϕ*s. For instance, SARS‐CoV‐2 is shown to more readily infect classically activated M1 M*ϕ*s,^68^ while hepatitis B virus (HBV) is more prone to infect alternatively activated M2 M*ϕ*s.^143^ Therefore, in order to obtain PSRM*ϕ*s susceptible or resistance to specific pathogens, generating M1 or M2 PSRM*ϕ*s directly through genetic modification may be a viable approach.^146^ Notably, iPSC‐derived chimeric antigen receptor (CAR)‐expressing M*ϕ*s (CAR‐iPSCdM*ϕ*s) have been developed and show antigen‐dependent polarization towards the M1 M*ϕ*s.^147^, ^148^ This provides new insights for utilizing PSRM*ϕ*s in studying the infection and clearance of extracellular pathogens.

However, there are still some challenges in gene editing in PSCs. Compared with tumour cell lines and mESCs, the efficiency of gene editing in hPSCs is below expectation.^149^ Even though high efficiency of gene editing is achieved, most hPSCs are killed by the Cas9‐induced toxicity, and p53 mutations are accumulated in successfully edited PSC clones.^150^, ^151^, ^152^ Furthermore, off‐targeting of gene editing remains to be addressed.^153^ Of note, novel gene editing system, such as base editors and prime editor, along with effective delivery system and small molecules may improve the editing efficiencies and simultaneously alleviate the off‐targeting.^153^ In generating genetically modified PSRM*ϕ*s, multiple genes may need to be inserted or deleted, which will pose higher requirements for gene editing.

Overall, PSCs are the valuable platforms for engineering PSRM*ϕ*s for specific applications, which can support or reduce the infections of certain pathogens by regulating the biological characteristics of PSRM*ϕ*s. However, more research is needed to develop mature gene editing systems for PSCs, and the modifications in PSCs must be undertaken with caution to avoid potential adverse effects, such as increased non‐specific infections or cytotoxicity.

## 5 CONCLUSIONS AND PERSPECTIVES

Infectious diseases pose significant threats to human and animal health, which has been particularly evident in the aftermath of the COVID‐19 pandemic. Here, we propose the potential of PSCs as the platform for genetic manipulation to create PSRM*ϕ*s, which may represent a novel and valuable cellular resource for studying infectious diseases targeting M*ϕ*s. While we have identified some challenges in creating PSRM*ϕ*s, numerous additional concerns remain, necessitating comprehensive and in‐depth exploration. Advanced platforms and technologies should be employed to enable PSRM*ϕ* differentiation. For example, virtual screening could be applied to identify the combinations of recombinant proteins and small molecule chemicals that facilitate PSC culture and PSRM*ϕ* differentiation. Moreover, co‐culturing M*ϕ*s with organoids could accurately replicate the in vivo cell‐to‐cell interactions, thereby enhancing the precision of experimental results.

Actually, PSRM*ϕ*s have enormous application potential in far more fields. In basic research, PSRM*ϕ*s may offer valuable insights into M*ϕ* development and lineage switching.^136^, ^154^ Given the roles of M*ϕ* polarization in autoimmune diseases,^155^ PSRM*ϕ*s may serve as the valuable platform for dissecting the mechanism of systemic lupus erythematosus, inflammatory bowel diseases, autoimmune myocarditis and autoimmune neuritis. In clinical application, PSRM*ϕ*s can be applied in cellular and gene therapies for cancer treatment. With almost indefinite proliferation capacity, PSRM*ϕ*s may provide unlimited access to CAR‐expressing M*ϕ*s to eliminate cancer cells.^147^, ^148^ In conclusion, although challenges remain, PSRM*ϕ*s will revolutionize both basic research and clinical application in the near future.

## AUTHOR CONTRIBUTIONS

Conceptualization, H.‐J.Q. L.‐F.L. and Z.L.; writing—original draft preparation, D.P.; writing—review and revision, M.L., Z.Y., T.Y., M.Y. and S.L.; figure preparation, D.P.; manuscript revision and supervision, H.‐J.Q., L.‐F.L. and Z.L.; and funding acquisition, L.‐F.L. All authors have read and agreed to the published version of the manuscript.

## CONFLICT OF INTEREST STATEMENT

The authors declare that the research was conducted in the absence of any commercial or financial relationships that could be construed as a potential conflict of interest.
